# Echocardiographic findings in young adults with primary hypertension: A scoping review protocol

**DOI:** 10.1371/journal.pone.0331460

**Published:** 2025-09-08

**Authors:** Lyana Shahirah Mohamad Yamin, Maryam Alsharqi, Mohd Asyiq Al-Fard Mohd Raffali, Akmal Sabarudin, Afifah Binti Mohamed

**Affiliations:** 1 Centre for Diagnostic, Therapeutic and Investigative Studies, Faculty of Health Sciences, Universiti Kebangsaan Malaysia, Kuala Lumpur, Malaysia; 2 Centre for Medical Imaging Studies, Faculty of Health Sciences, Universiti Teknologi MARA, Selangor, Malaysia; 3 Cardiac Technology Department, College of Applied Medical Sciences, Imam Abdulrahman Bin Faisal University, Dammam, Saudi Arabia; 4 Department of Medicine, Hospital Canselor Tuanku Muhriz, Faculty of Medicine, Universiti Kebangsaan Malaysia, Kuala Lumpur, Malaysia; Kasturba Medical College Manipal, INDIA

## Abstract

Echocardiography is the primary imaging tool for evaluating cardiac structure and function in patients with primary hypertension. A significant limitation of the current literature is that most studies focus on older adults, leaving a significant gap in understanding the cardiac effects of primary hypertension in young adults. This scoping review protocol aims to assess conventional echocardiographic parameters, left ventricular geometric patterns, and advanced echocardiographic findings for the early detection of cardiac changes in young adults aged 18–39 with primary hypertension. A systematic literature search will be conducted across the MEDLINE, EMBASE, Web of Science, Scopus, and Cochrane Central databases. This involves systematically reviewing existing literature to outline the reported echocardiographic findings, the parameters analysed, and the abnormalities identified within this population. It will establish the foundation for future reviews and primary research, supporting the evaluation of the diagnostic and prognostic significance of these findings for early detection and risk assessment, ranging from standard conventional measures to more advanced methods.

## Introduction

The global report on hypertension, titled “The Race Against a Silent Killer” (2023), highlights the significant impact of hypertension as a leading cause of mortality worldwide [1]. One in three adults affected is often asymptomatic, and nearly half of those affected are in an underdiagnosed situation due to the lack of healthcare interaction at a young age [2]. Hypertension prevalence has been steadily increasing in young adults, a population group traditionally considered at low risk for hypertension and its associated complications [3,4]. This shift is marked by a rise in smoking, excessive alcohol consumption, a growing prevalence of obesity, and a notable increase in salt intake, all of which stand as significant risk factors for various health issues [5]. Young adults suffering from hypertension experience the lowest blood pressure control rates when compared to older adults. This disparity is mainly attributed to inadequate documentation of lifestyle counselling and considerable delays in starting initial antihypertensive medications [6]. Additionally, a study of adults aged 30 and older showed a significant lack of awareness regarding their blood pressure levels, with only 50% of participants aware of their hypertension status [7].

The early development of hypertension in young adults may result in severe damage to target organs if not identified and managed appropriately [8]. Several studies have suggested that the onset of hypertension during childhood or adolescence may have a more significant impact on left ventricular (LV) remodeling and cardiovascular outcomes compared to the development of hypertension in adulthood [9,10]. Additionally, age-related physiological changes, such as alterations in vascular compliance and cardiac remodeling, may influence the manifestation and progression of hypertensive heart disease [11,12]. Agbaje et al. [13] stated that over the 7-year observation period, the occurrence of high systolic and diastolic blood pressure, left ventricular hypertrophy (LVH), and high LV filling pressure (hiLVFP) doubled among males and females.

Echocardiography is a non-invasive, radiation-free, and relatively inexpensive imaging modality that can be used to evaluate the impact of primary hypertension on cardiac structure and function, utilizing techniques such as two-dimensional (2D) imaging and strain imaging [14–16]. Echocardiography, while considered a second-line examination for hypertensive patients, reveals numerous indicators of poor prognosis linked to hypertension [17]. It is well-suited for serial scans that enable a real-time assessment of cardiac remodeling progression over time [18] and is ideal for detecting subclinical manifestations of hypertensive heart disease [19]. The key structural changes that can be monitored include LV mass index (LVMI), LVH, chamber dilation, relative wall thickness (RWT), left atrial (LA) volume, and aortic root dimensions [20]. In addition, echocardiography can detect systolic changes through ejection fraction (EF), tissue velocities (s’), and strain analysis, which depict early subclinical systolic dysfunction [21]. Diastolic dysfunction is quantifiable through transmitral flow parameters, such as the E/A ratio and E-wave deceleration time [22].

Furthermore, the advancement of echocardiography techniques such as two-dimensional speckle tracking echocardiography (2D-STE) provides a comprehensive reconstruction of the spatial deformation of the LV myocardium across various planes [23,24]. Due to its higher sensitivity, 2D-STE can identify subclinical myocardial dysfunction before conventional parameters show abnormalities, which is a key advantage in young patients, as early intervention can prevent progression [25,26]. Unlike tissue Doppler imaging, 2D-STE is not angle-dependent, which makes it more consistent and reproducible, regardless of the operator or centre [27]. Global longitudinal strain (GLS) from 2D-STE can also identify systolic dysfunction even when EF appears normal, helping identify young at-risk individuals who might benefit from intensive treatment [21]. In contrast to M-mode and 2D echocardiography, which have several limitations, 3D echocardiography provides potentially more precise measurements [28]. Real-time 3D measurement showed better agreement compared to M-mode or 2D LV mass calculations and exhibited less variability. Due to these benefits, 3D echocardiography is considered the preferred method for assessing LVH regression with antihypertensive treatments [17].

### Rationale

The 2023 European Society of Hypertension (ESH) Guidelines for Managing Arterial Hypertension highlighted that while most outcome-based randomized controlled trials included patients aged 18 and older, data on younger individuals have been consistently limited [29]. Establishing detailed and specific risk assessment charts tailored for younger populations is essential, as the existing Systematic Coronary Risk Evaluation Model Two (SCORE2) system restricts its risk quantification capabilities to individuals aged 40 years and older [29,30]. This limitation highlights the need for robust methodologies that accurately reflect the risk profiles of younger individuals, thereby facilitating more proactive healthcare interventions.

The alarming trend of increasing the prevalence of hypertension in young adults emphasizes an urgent need for comprehensive scientific exploration, particularly utilizing advanced imaging techniques to understand its implications. Integrating diverse research methodologies through a scoping review approach can significantly enhance the synthesis of evidence surrounding hypertension in this age group. This will enable an in-depth analysis of various echocardiographic imaging protocols and assist in standardizing parameter definitions crucial for consistent evaluation and comparison. Furthermore, it will provide a holistic overview of current knowledge regarding hypertension among young adults. Therefore, this scoping review intends to assess conventional echocardiographic parameters, left ventricular geometric patterns, and advanced echocardiographic findings for the early detection of cardiac changes in young adults with primary hypertension.

**Main research question:**

What are the common characteristic patterns of echocardiographic findings in young adults (ages 18–39) with primary hypertension?

**Sub-questions:**

1. What are the conventional echocardiographic parameters and geometric patterns of the left ventricles in young adults with primary hypertension?
2. What advanced echocardiographic measures of 2D-STE and 3D volumetric analysis are used to detect subclinical cardiac dysfunction in young adults with primary hypertension?

## Methods

This scoping review will adhere to the guidelines established by the PRISMA-SCR [31]. Following this guideline can ensure that all relevant information is meticulously documented, facilitating transparency, reproducibility, and critical appraisal of the research outcomes [32]. While developing this scoping review protocol, the Preferred Reporting Items for Systematic Reviews and Meta-analysis Protocols 2015 (PRISMA-P 2015) checklist [33] was utilized. This checklist serves as a comprehensive guide to ensure that all essential elements and requirements are systematically included in the protocol. Furthermore, this study has been officially registered with the Open Science Framework (OSF), a platform dedicated to increasing transparency in research practices. Researchers and interested parties can access the study details and its registration through the following link: https://osf.io/9cnvu.

### Eligibility criteria

This research framework will focus on conducting original studies to investigate the echocardiographic parameters observed in young adults with primary hypertension. It is strongly advised that the elements of the Population, Concept, and Context (PCC) methodology be thoroughly integrated [32]. **Participants:** Young adults, specifically those aged from 18 to 39 years. The age range mentioned in this context has been carefully selected according to the definitions established by the 2024 European Society of Cardiology (ESC) guideline; young adults are classified as individuals between the ages of 18–40 years [34] and the guidelines set by the ESH identify young patients as those who are under 40 years of age [29]. Therefore, for the purpose of this study, young adults are defined as individuals aged from 18 to 39 years. This classification is intentional, ensuring that the data and research findings specifically target the demographic of young adults. **Concept**: Only original research studies providing quantitative transthoracic echocardiographic (TTE) data will be eligible for inclusion. Studies must report measurable parameters essential for assessing LV mass, geometry, structure, and function in young adults with primary hypertension. The review will include studies using conventional 2D echocardiography, 2D-STE, 3D echocardiography, or any combination of these modalities. Both studies employing only standard echocardiographic techniques and those incorporating advanced imaging methods will be included to provide comprehensive coverage of available evidence, provided they follow established guidelines from the European Association of Cardiovascular Imaging (EACVI) and/or the American Society of Echocardiography (ASE) [35]. **Context**: This scoping review will include studies that explicitly identify their population as having “primary” or “essential” hypertension, or those that systematically exclude secondary causes of hypertension. To ensure population homogeneity, participants must not have uncontrolled cardiovascular diseases (heart failure, significant valvular disease, coronary artery disease, or significant arrhythmias requiring treatment), confirmed secondary hypertension causes (renal artery stenosis, primary aldosteronism, pheochromocytoma, coarctation of aorta, or Cushing’s syndrome), chronic kidney disease, conditions independently affecting cardiac structure (congenital heart disease, active malignancy, or severe liver disease), or pregnancy, regardless of current antihypertensive treatment status. This criterion is essential because these conditions can either cause secondary hypertension or independently alter cardiac structure and function, potentially confounding echocardiographic findings specific to primary hypertension. Studies with unclear or insufficient information about hypertension classification will be excluded unless clarification can be obtained from the authors’ contact.

**The source of evidence.** This review will be confined to published research papers from 2000 to 2025. A rigorous set of exclusion criteria has been meticulously defined to maintain the integrity and relevance of the findings. Any study that meets the following exclusion criteria will be systematically eliminated from consideration: (a) Studies with animal subjects will be excluded to ensure that only human-related research contributes to the findings (b) Non-research materials such as book chapters, commentaries, case reports, editorials, and literature reviews will not be included, as these do not provide original research data (c) Publications that are not in the English language will be excluded to maintain uniformity and comprehensibility in the data analysis (d) Any studies that involve participants diagnosed with medical conditions other than primary hypertension will also be excluded to focus exclusively on the effects and implications pertinent to this specific condition.

### Information source

A comprehensive and tailored literature search will be conducted across multiple electronic databases, including MEDLINE, EMBASE, Web of Science, Scopus, and the Cochrane Central databases. These databases have been selected based on their capacity to provide comprehensive citation sources. They cover an extensive range of subjects, including but not limited to epidemiology, pharmacology, public health policy, clinical practices, and health outcomes research, all of which are crucial for advancing knowledge and facilitating evidence-based practices in health-related research and inquiry [36].

### Search strategy

An extensive and systematic approach will be employed, utilizing a carefully selected combination of relevant keywords and Medical Subject Headings (MeSH) terms specifically related to primary hypertension, echocardiography, and young adults. This approach will ensure a thorough and inclusive literature review, facilitating the identification of relevant studies, articles, and research findings. By focusing on these specific areas, the literature inclusion process will be more targeted and dependable, leading to a solid understanding of the topic being studied. The search strategy will integrate the following terms:

#### Population-specific terms:.

(“young adults” OR “early adulthood” OR “young-onset”)

#### Context-specific terms:.

(“echocardiography” OR “transthoracic echocardiography” OR “cardiac imaging” OR “cardiac ultrasound” OR “speckle tracking echocardiography” OR “strain imaging” OR “2D-STE” OR “3D echocardiography” OR “three-dimensional echocardiography”)

#### Condition-specific terms:.

(“hypertension” OR “high blood pressure” OR “elevated blood pressure” OR “essential hypertension” OR “primary hypertension”)

The initial pilot search was conducted using the MEDLINE database through the PubMed interface. The outcome of this search, utilizing a specific search string designed to yield relevant articles, is outlined below:

*(“young adults” OR “early adulthood” OR “young-onset”) AND (“echocardiography” OR “transthoracic echocardiography” OR “cardiac imaging” OR “cardiac ultrasound” OR “speckle tracking echocardiography” OR “strain imaging” OR “2D-STE” OR “3D echocardiography” OR “three-dimensional echocardiography”) AND (“hypertension” OR “high blood pressure” OR “elevated blood pressure” OR “essential hypertension” OR “primary hypertension”)*

### Selection of source and the data charting process

Two reviewers will comprehensively and systematically screen titles and abstracts from articles using the Rayyan.ai platform. This process ensures that only the most relevant and high-quality research is considered for further evaluation. To uphold the review's integrity, the reviewers will employ a blind review methodology, thereby maintaining objectivity and minimizing potential bias in their evaluations.

To determine whether to include or exclude papers from consideration, the reviewers will rely on specific, pre-established inclusion and exclusion criteria that have been rigorously defined prior to the review process. In instances where disagreements arise between the reviewers concerning the suitability of certain articles, these discrepancies will be addressed through a discussion to facilitate consensus-building, allowing the reviewers to reach a mutual agreement based on logical reasoning and evidence.

After completing the initial screening and selecting the relevant articles, one reviewer will be responsible for carefully extracting essential data from these articles and entering it into Microsoft Excel. This data extraction process is crucial as it establishes the basis for subsequent analyses and conclusions in the research project.

### Data summarization and presentation

The data will be organized in a structured table to highlight key study characteristics and features. Table 1, for example, will list the authors, publication year, study design, age range, and gender. It will also specify the classification criteria for hypertension used across studies, indicating whether they rely on systolic and diastolic thresholds set by standard guidelines, as well as the duration of hypertension.

**Table 1:** Study Characteristics and Demographics.

**Author**	**Year**	**Study Design**	**Age Range (year)**	**Gender**	**HTN Definition (mmHg)**	**HTN Duration (year)**

HTN, hypertension.

In the detailed mass and structural analysis, key parameters will be tabulated as in Table 2, which may comprise interventricular septum (IVS) thickness, LV internal diastolic diameter (LVIDD), LV internal systolic diameter (LVISD), LV posterior wall thickness (LVPW), LVMI, RWT, percentage of LVH, and left atrial volume index (LAVI). Two main indexing methods for LVMI will also be noted: Body surface area-based (LVMI-BSA) and height-based (LVMI-H^2.7^) indexing.

**Table 2:** Analysis of Mass and Structural Parameters.

**Author**	**IVS (mm)**	**LVIDD (mm)**	**LVISD (mm)**	**LVPW (mm)**	**RWT**	**LVH (%)**	**LAVI (mL/m**^**2**^)	**LVM (g)**	**LVMI- BSA (g/m**^**2**^)	**LVMI - Height**^**2.7**^ **(g/m**^**2.7**^)	**Geometric Pattern**

IVS, interventricular septum; LVIDD, left ventricular internal diastolic diameter; LVISD, left ventricular internal systolic diameter; LVPW, left ventricular posterior wall thickness; RWT, relative wall thickness; LVH, left ventricular hypertrophy; LAVI, left atrial volume index; LVM, left ventricular mass; LVMI-BSA, left ventricular mass indexed with body surface area-based; LVMI-Height^2.7^, left ventricular mass indexed with height^2.7^.

^a^All values will be presented as mean ± standard deviation, percentage where applicable.

Subsequent tables will further explore the assessment of echocardiographic parameters, focusing on basic and advanced structural and functional measures. For the basic echocardiographic parameters, key systolic measures, including EF, and diastolic indices, such as early diastolic filling velocity and deceleration time, will be listed in Table 3.

**Table 3:** Conventional Echocardiographic Parameters.

**Author**	**EF (%)**	**FS (%)**	**E/A Ratio**	**DT (ms)**	**IVRT (ms)**	**e’ Lateral (cm/s)**	**E/e’ Ratio**

EF, ejection fraction; FS, fractional shortening; DT, deceleration time; IVRT, isovolumic relaxation time; e’, e-prime wave; E/e’, E-wave velocity divided by the lateral e-prime velocity.

^a^All values will be presented as mean ± standard deviation, percentage where applicable.

In addition to the standard parameters, Table 4 will present data on advanced echocardiographic parameters, including the 2D-STE percentage of GLS, global circumferential strain (GCS), global radial strain (GRS), and the 3D volumetric index (3DVi) and 3D-EF percentage. These advanced measures are essential to understanding both the physiological and pathological changes in young adults with hypertension, providing a holistic view of cardiac health within this demographic.

**Table 4:** Advanced Echocardiographic Parameters.

**Author**	**2D-STE (%)**	**3DVi (ml/m**^**2**^)	**3D-EF (%)**

2D-STE, two-dimensional speckle tracking echocardiography; 3DVi, three-dimensional volumetric index; 3D-EF, three-dimensional ejection fraction.

^a^All values will be presented as mean ± standard deviation, percentage where applicable.

## Discussion

The traditional assessment of systolic function has relied predominantly on EF, which remains a crucial parameter for detecting overt systolic dysfunction. However, growing evidence demonstrates that EF lacks sensitivity for identifying early myocardial impairment, particularly in the context of hypertensive heart disease, where systolic dysfunction may be subtle and masked by preserved overall ventricular function [35]. GLS has emerged as a revolutionary parameter that addresses this limitation by quantifying myocardial deformation with superior sensitivity compared to conventional measures. Studies consistently demonstrate that GLS can detect subclinical systolic dysfunction in hypertensive patients with preserved EF, with values less negative than −18% indicating early myocardial involvement [21,26]. This enhanced sensitivity makes GLS particularly valuable for identifying young adults at risk for progression to overt heart failure.

The LVMI and RWT represent basic measurements for characterizing the structural cardiac response to chronic hypertension. These parameters enable precise classification of LV geometric patterns, distinguishing between normal geometry, concentric remodeling, concentric hypertrophy, and eccentric hypertrophy [37]. The clinical significance of these geometric patterns extends beyond mere morphological description, as they carry distinct prognostic implications and guide therapeutic decision-making. Concentric remodeling and hypertrophy, characterized by increased RWT with or without elevated LV mass, reflect the heart's adaptive mechanism to sustained pressure overload [38] and are associated with increased cardiovascular risk, even in the absence of overt clinical symptoms [39].

Evaluating diastolic function is crucial in hypertensive heart disease, as diastolic abnormalities often represent the earliest manifestation of cardiac involvement, which typically appears years before systolic dysfunction [22]. The E/e’ ratio serves as a reliable non-invasive estimate of LV filling pressures, with elevated values indicating increased LA pressure and impaired ventricular relaxation. The LAVI provides complementary information by reflecting the chronic hemodynamic burden imposed by elevated filling pressures over time [40]. An enlarged LA, as quantified by LAVI, serves as a morphological marker of chronic diastolic dysfunction and correlates with adverse cardiovascular outcomes. The combination of these parameters enables a comprehensive assessment of diastolic function and helps identify patients with heart failure with preserved EF, a condition particularly prevalent among hypertensive individuals.

2D-STE and 3D volumetric analysis represent the cutting edge of cardiac imaging technology, offering unprecedented insights into myocardial mechanics and chamber geometry [18]. These advanced techniques can potentially detect subtle abnormalities that conventional echocardiographic methods may miss, thus facilitating the earlier identification of cardiac involvement in young hypertensive adults. Speckle tracking technology enables a comprehensive assessment of myocardial strain patterns, extending beyond GLS to include circumferential and radial strain components. This provides a more complete picture of ventricular function. 3D echocardiography offers superior accuracy in chamber quantification and may reveal geometric abnormalities not apparent on 2D imaging [20].

This comprehensive approach to parameter selection ensures that our scoping review will capture the full spectrum of echocardiographic findings relevant to the early detection of primary hypertensive heart disease among young adults, ranging from established conventional measures to emerging advanced techniques that may define the future of cardiac assessment in this population.

## Limitation

The search strategy will be limited to a predetermined set of databases. This targeted approach strikes a balance between comprehensiveness and feasibility, enabling thorough coverage within available resources and time constraints. By focusing on high-quality, relevant databases rather than attempting an exhaustive search across all available sources, this method optimizes both the precision and manageability of the literature review process.

## Supporting information

S1 File Preferred Reporting Items for Systematic Reviews and Meta-analyses for Protocols 2015 (PRISMA-P 2015) Checklist. (DOCX)

## References

[1] Global report on hypertension: the race against a silent killer. Geneva: World Health Organization; 2023. Licence: CC BY-NC-SA 3.0 IGO. Available from: https://www.who.int/publications/i/item/9789240081062

[2] Unger T, Borghi C, Charchar F, Khan NA, Poulter NR, Prabhakaran D, et al. 2020 International Society of Hypertension Global Hypertension Practice Guidelines. Hypertension. 2020;75(6):1334–57. doi: 10.1161/HYPERTENSIONAHA.120.15026 32370572

[3] Yano Y, Stamler J, Garside DB, Daviglus ML, Franklin SS, Carnethon MR, et al. Isolated systolic hypertension in young and middle-aged adults and 31-year risk for cardiovascular mortality: the Chicago Heart Association Detection Project in Industry study. J Am Coll Cardiol. 2015;65(4):327–35. doi: 10.1016/j.jacc.2014.10.060 25634830 PMC4948287

[4] Niiranen TJ, Kalesan B, Hamburg NM, Benjamin EJ, Mitchell GF, Vasan RS. Relative Contributions of Arterial Stiffness and Hypertension to Cardiovascular Disease: The Framingham Heart Study. JAHA. 2016;5(11). doi: 10.1161/jaha.116.004271 PMC5210358 27912210

[5] Meher M, Pradhan S, Pradhan SR. Risk Factors Associated With Hypertension in Young Adults: A Systematic Review. Cureus. 2023;15(4):e37467. doi: 10.7759/cureus.37467 37187665 PMC10181897

[6] Xu L, Wang N, Chen X, Liang Y, Zhou H, Yan J. Quantitative evaluation of myocardial layer-specific strain using two-dimensional speckle tracking echocardiography among young adults with essential hypertension in China. Medicine (Baltimore). 2018;97(39):e12448. doi: 10.1097/MD.0000000000012448 30278524 PMC6181480

[7] Abdul-Razak S, Daher AM, Ramli AS, Ariffin F, Mazapuspavina MY, Ambigga KS, et al. Prevalence, awareness, treatment, control and socio demographic determinants of hypertension in Malaysian adults. BMC Public Health. 2016;16(1). doi: 10.1186/s12889-016-3008-y PMC4839122 27097542

[8] Diaz KM, Shimbo D. Physical activity and the prevention of hypertension. Curr Hypertens Rep. 2013;15(6):659–68. doi: 10.1007/s11906-013-0386-8 24052212 PMC3901083

[9] Chen X, Wang Y. Tracking of blood pressure from childhood to adulthood: a systematic review and meta-regression analysis. Circulation. 2008;117(25):3171–80. doi: 10.1161/CIRCULATIONAHA.107.730366 18559702 PMC3568631

[10] Aatola H, Hutri-Kähönen N, Juonala M, Viikari JSA, Hulkkonen J, Laitinen T, et al. Lifetime risk factors and arterial pulse wave velocity in adulthood: the cardiovascular risk in young Finns study. Hypertension. 2010;55(3):806–11. doi: 10.1161/HYPERTENSIONAHA.109.145102 20083727

[11] Laurent S, Cockcroft J, Van Bortel L, Boutouyrie P, Giannattasio C, Hayoz D, et al. Expert consensus document on arterial stiffness: methodological issues and clinical applications. Eur Heart J. 2006;27(21):2588–605. doi: 10.1093/eurheartj/ehl254 17000623

[12] Fleg JL, Strait J. Age-associated changes in cardiovascular structure and function: a fertile milieu for future disease. Heart Fail Rev. 2012;17(4–5):545–54. doi: 10.1007/s10741-011-9270-2 21809160 PMC4677819

[13] Agbaje AO. Elevated Blood Pressure and Worsening Cardiac Damage During Adolescence. J Pediatr. 2023;257:113374. doi: 10.1016/j.jpeds.2023.02.018 36870560

[14] Thomas JD, Popović ZB. Assessment of left ventricular function by cardiac ultrasound. J Am Coll Cardiol. 2006;48(10):2012–25. doi: 10.1016/j.jacc.2006.06.071 17112991

[15] Mor-Avi V, Lang RM, Badano LP, Belohlavek M, Cardim NM, Derumeaux G, et al. Current and evolving echocardiographic techniques for the quantitative evaluation of cardiac mechanics: ASE/EAE consensus statement on methodology and indications endorsed by the Japanese Society of Echocardiography. J Am Soc Echocardiogr. 2011;24(3):277–313. doi: 10.1016/j.echo.2011.01.015 21338865

[16] Smiseth OA, Torp H, Opdahl A, Haugaa KH, Urheim S. Myocardial strain imaging: how useful is it in clinical decision making? Eur Heart J. 2015;37(15):1196–207. doi: 10.1093/eurheartj/ehv529 26508168 PMC4830908

[17] Lee J-H, Park J-H. Role of echocardiography in clinical hypertension. Clin Hypertens. 2015;21:9. doi: 10.1186/s40885-015-0015-8 26893921 PMC4750785

[18] Galderisi M, Cosyns B, Edvardsen T, Cardim N, Delgado V, Di Salvo G, et al. Standardization of adult transthoracic echocardiography reporting in agreement with recent chamber quantification, diastolic function, and heart valve disease recommendations: an expert consensus document of the European Association of Cardiovascular Imaging. Eur Heart J Cardiovasc Imaging. 2017;18(12):1301–10. doi: 10.1093/ehjci/jex244 29045589

[19] Oh JK, Park J-H. Role of strain echocardiography in patients with hypertension. Clin Hypertens. 2022;28(1). doi: 10.1186/s40885-021-00186-y PMC8845306 35164856

[20] Lang RM, Badano LP, Mor-Avi V, Afilalo J, Armstrong A, Ernande L, et al. Recommendations for Cardiac Chamber Quantification by Echocardiography in Adults: An Update from the American Society of Echocardiography and the European Association of Cardiovascular Imaging. Eur Heart J Cardiovasc Imaging. 2015;16(3):233–71. doi: 10.1093/ehjci/jev014 25712077

[21] Yingchoncharoen T, Agarwal S, Popović ZB, Marwick TH. Normal ranges of left ventricular strain: a meta-analysis. J Am Soc Echocardiogr. 2013;26(2):185–91. doi: 10.1016/j.echo.2012.10.008 23218891

[22] Nagueh SF, Smiseth OA, Appleton CP, Byrd BF, Dokainish H, Edvardsen T, et al. Recommendations for the Evaluation of Left Ventricular Diastolic Function by Echocardiography: An Update from the American Society of Echocardiography and the European Association of Cardiovascular Imaging. J Am Soc Echocardiogr. 2016;29: 277–314. Available from: doi: 10.1016/j.echo.2016.01.011 27037982

[23] Teske AJ, De Boeck BWL, Melman PG, Sieswerda GT, Doevendans PA, Cramer MJM. Echocardiographic quantification of myocardial function using tissue deformation imaging, a guide to image acquisition and analysis using tissue Doppler and speckle tracking. Cardiovasc Ultrasound. 2007;5:27. doi: 10.1186/1476-7120-5-27 17760964 PMC2000459

[24] Cameli M, Mandoli GE, Sciaccaluga C, Mondillo S. More than 10 years of speckle tracking echocardiography: Still a novel technique or a definite tool for clinical practice? Echocardiography. 2019;36:958–70. doi: 10.1111/echo.14339 30974002

[25] Collier P, Phelan D, Klein A. A Test in Context: Myocardial Strain Measured by Speckle-Tracking Echocardiography. J Am Coll Cardiol. 2017;69(8):1043–56. doi: 10.1016/j.jacc.2016.12.012 28231932

[26] Kalam K, Otahal P, Marwick TH. Prognostic implications of global LV dysfunction: a systematic review and meta-analysis of global longitudinal strain and ejection fraction. Heart. 2014;100(21):1673–80. doi: 10.1136/heartjnl-2014-305538 24860005

[27] Geyer H, Caracciolo G, Abe H, Wilansky S, Carerj S, Gentile F, et al. Assessment of myocardial mechanics using speckle tracking echocardiography: fundamentals and clinical applications. J Am Soc Echocardiogr. 2010;23(4):351–69; quiz 453–5. doi: 10.1016/j.echo.2010.02.015 20362924

[28] Cacciapuoti F. Echocardiographic Evaluation of Ejection Fraction: 3-DE Versus 2-DE and M-Mode. 2008. Available from: https://journals.lww.com/hrtv/fulltext/2008/09020/echocardiographic_evaluation_of_ejection_fraction_.5.aspx

[29] Mancia G, Kreutz R, Brunström M, Burnier M, Grassi G, Januszewicz A, et al. 2023 ESH Guidelines for the management of arterial hypertension The Task Force for the management of arterial hypertension of the European Society of Hypertension: Endorsed by the International Society of Hypertension (ISH) and the European Renal Association (ERA). J Hypertens. 2023;41(12):1874–2071. doi: 10.1097/HJH.0000000000003480 37345492

[30] Tokgozoglu L, Torp-Pedersen C. Redefining cardiovascular risk prediction: is the crystal ball clearer now?. European Heart Journal. 2021;42(25):2468–71. doi: 10.1093/eurheartj/ehab310 34120165

[31] Tricco AC, Lillie E, Zarin W, O’Brien KK, Colquhoun H, Levac D, et al. PRISMA Extension for Scoping Reviews (PRISMA-ScR): Checklist and Explanation. Ann Intern Med. 2018;169(7):467–73. doi: 10.7326/M18-0850 30178033

[32] Hadie SNH. ABC of a Scoping Review: A Simplified JBI Scoping Review Guideline. EIMJ. 2024;16(2):185–97. doi: 10.21315/eimj2024.16.2.14

[33] Moher D, Shamseer L, Clarke M, Ghersi D, Liberati A, Petticrew M, et al. Preferred reporting items for systematic review and meta-analysis protocols (PRISMA-P) 2015 statement. Syst Rev. 2015;4(1). doi: 10.1186/2046-4053-4-1 PMC4320440 25554246

[34] McEvoy JW, McCarthy CP, Bruno RM, Brouwers S, Canavan MD, Ceconi C, et al. 2024 ESC Guidelines for the management of elevated blood pressure and hypertension. Eur Heart J. 2024;45(38):3912–4018. doi: 10.1093/eurheartj/ehae178 39210715

[35] Marwick TH, Gillebert TC, Aurigemma G, Chirinos J, Derumeaux G, Galderisi M, et al. Recommendations on the use of echocardiography in adult hypertension: a report from the European Association of Cardiovascular Imaging (EACVI) and the American Society of Echocardiography (ASE)†. Eur Heart J Cardiovasc Imaging. 2015;16(6):577–605. doi: 10.1093/ehjci/jev076 25995329

[36] Peters MD, Godfrey C, McInerney P, Munn Z, Tricco AC, Khalil H. Scoping reviews. JBI Manual for Evidence Synthesis. JBI; 2024. Available from: https://synthesismanual.jbi.global. https://doi.org/10.46658/JBIMES-24-09

[37] Gerdts E, Cramariuc D, de Simone G, Wachtell K, Dahlöf B, Devereux RB. Impact of left ventricular geometry on prognosis in hypertensive patients with left ventricular hypertrophy (the LIFE study). Eur J Echocardiogr. 2008;9(6):809–15. doi: 10.1093/ejechocard/jen155 18490279

[38] Gaasch WH, Zile MR. Left ventricular structural remodeling in health and disease: with special emphasis on volume, mass, and geometry. J Am Coll Cardiol. 2011;58(17):1733–40. doi: 10.1016/j.jacc.2011.07.022 21996383

[39] Verdecchia P, Schillaci G, Borgioni C, Ciucci A, Battistelli M, Bartoccini C, et al. Adverse prognostic significance of concentric remodeling of the left ventricle in hypertensive patients with normal left ventricular mass. J Am Coll Cardiol. 1995;25(4):871–8. doi: 10.1016/0735-1097(94)00424-o 7884090

[40] Tsang TSM, Barnes ME, Gersh BJ, Bailey KR, Seward JB. Left atrial volume as a morphophysiologic expression of left ventricular diastolic dysfunction and relation to cardiovascular risk burden. Am J Cardiol. 2002;90(12):1284–9. doi: 10.1016/s0002-9149(02)02864-3 12480035

